# Differential affects of low glucose on the macroheterogeneity and microheterogeneity of glycosylation in CHO-EG2 camelid monoclonal antibodies

**DOI:** 10.1186/1753-6561-7-S6-P112

**Published:** 2013-12-04

**Authors:** Bo Liu, Carina Villacres-Barragan, Erika Lattova, Maureen Spearman, Michael Butler

**Affiliations:** 1 Dept of Microbiology, University of Manitoba, Winnipeg, Manitoba, R3T 2N2, CA, USA; 2 Dept of Chemistry, University of Manitoba, Winnipeg, Manitoba, R3T 2N2, CA, USA

## Background

The demand for high yield recombinant protein production systems has focused industry on culture media and feed strategies that optimize productivity, yet maintain product quality attributes such as glycosylation. Minimizing media components such as glucose, reduces the production of lactate, but may also affect glycosylation. The first steps in the glycosylation pathway involve the synthesis of lipid-linked oligosaccharides (LLOs). Glycan macroheterogeneity is introduced by variation in site-specific glycosylation with the transfer of the oligosaccharide to the protein. Further modification of the oligosaccharide can occur through processing reactions, where some sugars are removed and additional sugars added. This produces microheterogeneity of the glycan pool. Both macroheterogeneity and microheterogeneity may be affected by fermentation conditions. The objective of this study has been to investigate the effect of variable concentrations of glucose on the glycosylation patterns of a camelid monoclonal antibody produced in Chinese hamster ovary (CHO) cells and to further evaluate their effect on components of the N-glycosylation pathway.

## Materials and methods

A CHO cell line recombinantly expressing chimeric antibodies EG2 with a camelid single domain fused to human Fc regions was used in this study. Cells were inoculated at 2.6 x 10^6^ cells/ml into 7 shake flasks (250 ml) each containing 80 ml of media with a different initial glucose concentration varying from 0 to 25 mM. The cultures were maintained and monitored under standard shaking conditions in an incubator over a 24 hr period.

Cells were harvested and quenched to stop any subsequent metabolic activities [1]. LLOs were extracted from the cells using a previously established method [2]. Mild acid cleaved glycans were labeled with 2-aminobenzamide and analyzed by high performance liquid chromatography (HPLC) using the technique of hydrophilic interaction liquid chromatography (HILIC). The structures were assigned using standard GU values from the GlycoBase database (NIBRT.ie) [3] and confirmed by Mass spectrometric analysis.

Antibodies were purified from culture supernatants with a Protein A affinity column and run under denaturing conditions on 8-16% SDS-PAGE gels and stained with Coomassie Brilliant Blue (CBB). The density ratio between upper and lower bands was determined by densitometry. The protein bands were removed by scalpel, washed, and treated with Peptide-N-Glycosidase F for 18 h to remove the attached glycans. MS analysis was carried out on the MALDI-TOF/TOF mass spectrometer to confirm aglycosylated Mabs in the lower band, and glycosylated proteins present in the upper band. The isolated N-linked glycans were labeled with 2-AB [4]. Glycan structures were assigned using standard GU values from HILIC analysis in GlycoBase. Structures were confirmed by exoglycosidase enzymatic digestion arrays according to method of Royle et al (2010).

## Results

**Figure 1:**
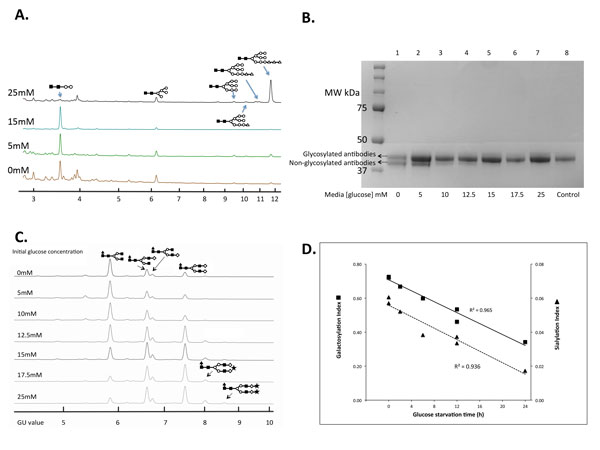
**The availability of glucose to CHO cells affects the intracellular lipid-linked oligosaccharide distribution, site occupancy and the N-glycosylation profile of a monoclonal antibody.A.** Lipid-linked oligosaccharide (LLO) profiles. The glycans from each sample were acid hydrolyzed from the lipid carriers, 2-AB labeled and detected by HILIC. (Glc Δ Man Ο and GlcNAc ?). **B**. Separation of EG2 antibodies on reduced 8-16% SDS-PAGE gel. The purified antibody in lane 8 was isolated from the culture prior to the 24 h incubation. Upper bands in lanes 1 to 4 correspond to glycosylated antibodies, and the lower bands were determined to be non-glycosylated antibodies. **C**. HPLC profiles of N-glycans isolated from EG2 antibodies produced by CHO cells with various initial glucose concentrations during a 24 h incubation. **D**. The effect of exposure time of cells to media depleted of glucose on the galactosylation (GI; ¦) and the sialylation (SI; ?) indices of EG2 antibodies produced by CHO cells.

Peaks corresponding to the LLOs from each of the previously described cultures with varying glucose concentration cultures were compared (Figure 1.A.). Samples from cultures containing 25mM glucose displayed a prominent large peak with a GU value of 11.7 representing 63% of the total LLOs and designated as the Glc3Man9GlcNAc2^a ^structure (Figure 1.A.). Small peaks were designated as Glc2Man9GlcNAc2, Glc1Man9GlcNAc2, Man9GlcNAc2, Man5GlcNAc2 and Man2GlcNAc2 structures. For cells grown at an initial glucose concentration of less than 15 mM the predominant peak was Man2GlcNAc2 with a significant level of the Man5GlcNAc2 structure but the percentage of the Glc3Man9GlcNAc2 structure was reduced significantly to 2.9% of the overall LLOs. It is important to note that these cultures (≤15mM glucose) were under conditions of glucose depletion for at least 4 h prior to harvest.

LLO with a completed glycan structure Glc3Man9GlcNAc2 is an essential precursor for N-glycosylation. Thus, the effect of glucose concentration on the macroheterogeneity and microheterogeneity of the fully formed glycoprotein were examined next. Protein A purified antibodies from cultures after 24 h were analyzed on reduced SDS-PAGE gels 1. B. The antibodies produced by cells grown in 17.5-25 mM glucose displayed one single strong band corresponding to the glycosylated heavy chain. Proteins isolated from cell culture with 15 mM initial glucose concentration (Lane 5) showed a faint band underneath the predominant gel band. The proportional density of the lower band in the 12.5 mM glucose sample was 26% which increased gradually to 52% for samples taken from cultures with no added glucose (Table 1). The lower protein bands were suspected to be deglycosylated proteins due to an estimated 2% weight loss, which corresponds to the typical mass of glycan found on IgGs [5]. Samples of antibody showing two gel bands were analyzed by MALDI-MS. This showed m/z values of 82,670 and 79,350 which are the expected masses of the glycosylated and non-glycosylated forms, respectively of the complete antibodies.

**Table 1:** Quantitative densitometry of Protein A purified EG2 antibodies stained with coomassie blue (*n *= 5).

Initial glucose concentration (mM)	% Glycosylated protein	% Non-glycosylation protein
0	48 ± 1	52 ± 1
5	60 ± 4	40 ± 4
10	69 ± 2	31 ± 2
12.5	74 ± 2	26 ± 2
15	100	0
17.5	100	0
25	100	0
Control	100	0

To compare the difference in glycosylation profiles of EG2 antibodies induced by various glucose concentrations, the glycans were released from the Protein A-purified Mabs with PNGase F, and analyzed by HILIC HPLC. The glycan pool was separated into six major peaks which eluted between 33 and 43 minutes with corresponding GU values between 5 and 9 (Figure 1. C.). Structures were provisionally assigned from GU values with reference to the Glycobase and confirmed by a series of exoglycosidase enzyme array digestions. This allowed the identification of biantennary glycan structures with variable galactosylation, fucosylation and sialylation. The predominant glycan structure of antibodies isolated from the 25 mM glucose culture was the fully galactosylated biantennary and fucosylated structure, Fuc(6)GlcNAc2Gal2 , which comprised 60% of the overall glycans. Fuc(6)GlcNAc2Gal0 and Fuc(6)GlcNAc2Gal2 structures were determined at 6% and 34%, respectively. The structures were found in samples from all cultures analyzed but there was a significant shift to lower galactosylation and sialylation in samples derived from cultures with lower glucose.

^a ^Glc, glucose; Man, mannose; GlcNAc, N-acetylglucosamine.

^b ^Fuc, fucose; Gal, galactose.

Each glycan pool was assigned a galactosylation index (GI) and a sialylation index (SI) based upon the relative peak areas on the HPLC profile. In this experiment the GI value changed from 0.35 to 0.72 as the availability of glucose increased for the cells. Sialylation is dependent upon prior galactosylation of a glycan and consequently shows lower values with corresponding SI values from 0.019 to 0.058. There was a strong positive correlation between the GI and SI value determined for each sample and the time spent by the corresponding cells in glucose deprived media over the 24 h experimental period (R2 = 0.965 and 0.936 for the GI and SI values respectively; Figure 1.D.).

## Conclusion

N-glycosylation is an important post-translation modification in mammalian cells, which is known to impact the quality and efficacy of therapeutic recombinant proteins. In this study, we focused on the effect of glucose concentration on several aspects in N-glycosylation pathways in CHO-EG2 cells. The depletion of glucose as the main carbohydrate source during cell culture, can reduce the capacity for N-glycosylation. Reduced availability of the full-length LLO precursor occurred by glucose deprivation and resulted in the accumulation of truncated dolichol linked glycans. This led to reduced glycosylation in the EG2 antibodies. Glucose deprivation also led to changes in microheterogeneity with a decrease in galactosylation and sialylation. It is concluded that low glucose concentrations in culture altered LLO synthesis and N-glycan profiles of the antibody.
